# Physiology and Aging: New Understanding of Organs That Affect the Physiology of Aging

**DOI:** 10.1093/geroni/igaa057.2641

**Published:** 2020-12-16

**Authors:** Daniela Drummond-Barbosa

## Abstract

As organisms age, many changes occur to their physiology, which in turn impact the function of multiple tissues. It is therefore critical to investigate the fundamental mechanisms of how endocrine organs shape our physiology, and how changes in our physiology affect stem cell lineages, which generate new cells for maintenance and repair of tissues/organs throughout life. This symposium will highlight the research in the laboratories of Dr. Gerard Karsenty (Columbia University) on the multiple endocrine functions of bone, of Dr. Nicholas Buchon (Cornell University) on the role of host-microbe interactions in intestinal homeostasis, of Dr. Jane Hubbard (NYU/Skirball Institute) on the physiological control of the germline, and of Dr. Daniela Drummond-Barbosa (Johns Hopkins University) on how diet and adipocyte factors regulate oogenesis. As research by these and other groups illustrate, the complex physiological regulation of tissue/organ maintenance and function is not only a fascinating biological problem, but it also has implications for many diseases and other conditions that are tightly linked to our endocrine state, including aging.

